# Velocity-Based Curve Differential Repeated Sprinting Training: A Novel Approach to Shape Physical Performance in Young Basketball Players

**DOI:** 10.3390/jfmk10020157

**Published:** 2025-05-03

**Authors:** Jorge Arede, Jack Wells, Mark Williams, Franc Garcia, Wolfgang Schöllhorn

**Affiliations:** 1 School of Education, Polytechnic Institute of Viseu, 3504-501 Viseu, Portugal; 2 Department of Sports, Exercise and Health Sciences, University of Trás-os-Montes and Alto Douro, 5001-801 Vila Real, Portugal; 3 Research Center in Sports Sciences, Health Sciences and Human Development, 5001-801 Vila Real, Portugal; 4 England Golf, National Golf Centre, Woodhall Spa LN10 6PU, UK; jack.wells@aru.ac.uk; 5 Department of Sport and Exercise Sciences, Anglia Ruskin University, Cambridge CB1 1PT, UK; 6 School of Sport, Rehabilitation and Exercise Sciences, University of Essex, Colchester CO4 3SQ, UK; mark.williams1@aru.ac.uk; 7 School of Psychology and Sport Science, Anglia Ruskin University, Cambridge CB1 1PT, UK; 8 Sports Performance Area, Futbol Club Barcelona, 08028 Barcelona, Spain; francgarciagarrido@gmail.com; 9 Barça Innovation Hub, Futbol Club Barcelona, 08028 Barcelona, Spain; 10 Grup de Recerca en Ciències de l’Esport INEFC Barcelona (GRCEIB), Institut Nacional d’Educació Física de Catalunya (INEFC), Universitat de Barcelona, 08038 Barcelona, Spain; 11 Institute of Sport Science, Training and Movement Science, University of Mainz, 55122 Mainz, Germany; wolfgang.schoellhorn@uni-mainz.de

**Keywords:** team sports, variation, movement variability, adolescence, bilateral asymmetry

## Abstract

**Background:** Basketball necessitates high-intensity, intermittent efforts with multidirectional and unpredictable movements, requiring training strategies that enhance players’ physical capacity to meet these demands efficiently. This study aimed to examine the effects of a velocity-based curve differential sprinting training on the overall performance of young male basketball players. **Methods:** A total of 24 participants were tested for single- and double-legged counter-movement jump (CMJ) height, 10 m linear sprint time, and agility performance in a pre–post–retention test design. The 12-week intervention consisted of two weekly sessions with differential sprint training in addition to normal basketball training. Sessions consisted of two sets of ten 20 m sprints whereby participants were instructed to perform additional fluctuations in joints velocity for each repetition. **Results:** Results show strong evidence for H_1_ (significant effect) for the experimental group in CMJ height (right leg) (BF_10_ = 19.24) between pre-, post-, and retention test values (BF_10_ = 10.24–17.85). For the remaining variables, no significant differences were observed. In contrast, the control group showed no significant effects including sprinting variables, indicating limited training effects. **Conclusions:** In conclusion, this research found that the 12-week differential curve sprinting training improved physical performance in the CMJ for the experimental group. Therefore, adding velocity-based random fluctuations during curve sprint training could be an effective training strategy for enhancing jumping performance in youth basketball players, which should encourage practitioners to implement different variations of the differential training approach.

## 1. Introduction

Basketball is a team sport that requires the simultaneous application of multiple technical, tactical, social, psychological, and physical abilities [1]. Physically, young basketball players cover between 82.5 and 87.1 m per minute [2]. In addition, players must execute high volumes of direction changes and associated accelerations and decelerations [3]. At the same time, players must perform technical skills, make decisions, and manage emotions [4]. Accordingly, basketball demands high-intensity, intermittent efforts that are multidirectional and unpredictable. Consequently, coaches and players must use training strategies that improve their physical capacity to cope with these demands more effectively [5].

Numerous training methods have been used in basketball to enhance young players’ physical development, with successful outcomes. These strategies include resistance training [6], plyometric exercises [7], eccentric overload training [8], and combined training protocols (e.g., complex training) [9]. However, these training methods often require material resources or training volume that makes them difficult to replicate in contexts with fewer resources, such as basketball gymnasiums. Consequently, alternative methods where only the body is included, such as repeated sprints and high-intensity interval training, have gained popularity in team sports [10]. The existing body of evidence has demonstrated the benefits of these strategies, which are relatively easy to implement and low-cost [10].

In this context, evaluating the effects of methods on physical performance outcomes that are easier to implement may help coaches and strength and conditioning practitioners enhance the physical capabilities of youth players. However, the training often deployed for such outcomes typically follows traditional approaches, overlooking recent innovations in motor learning. In this regard, one theory that has gained significant interest in sports is differential learning [11]. Differential learning is a method that encourages movement variability by incorporating varied repetitions that can always be different, and which occur without augmented feedback [12]. Variations in DL training include changes in movement geometry, velocity, accelerations, and rhythms [12]. In contrast to traditional training methods, this approach considers “errors” in movement execution to be potentially positive for the motor learning of skill, reflecting the individuality and non-linearity of development in each athlete [12]. Accordingly, this approach enhances athletes’ motor adaptability, increasing their ability to respond to team sports’ dynamic and unpredictable environments. Moreover, programs based on differential learning have extended beyond the development of physical skills to also enhance technical and tactical abilities, with highly positive outcomes [13,14].

The existing body of evidence supports the possibility that differential learning is a suitable and effective strategy, even when implemented with relatively low training volumes [11]. In the context of team sports, its effectiveness has primarily been tested in football, volleyball, and basketball [14,15,16,17,18,19]. For example, differential learning applied to the repeated sprint training using the movement geometry fluctuations (i.e., joint angles) has demonstrated enhanced physical capabilities in basketball. Specifically, a study conducted on female basketball players demonstrated that incorporating movement fluctuations during repeated sprints (2 × 10 repetitions of 20 m sprints) led to improvements in sprinting ability, counter-movement jump performance, and reduction in bilateral asymmetries [19]. Another training program with similar characteristics, conducted with male basketball players, also revealed positive outcomes. In particular, benefits were observed in sprint performance, especially among more mature athletes, as well as improvements in bilateral asymmetries for less mature athletes [17]. Furthermore, a recent study explored the effects of velocity- and acceleration-based (i.e., joint angular velocity and acceleration) differential plyometric training in young male basketball players, revealing significant improvements in bilateral counter-movement jump height and moderate gains in agility and unilateral jump performance [20]. However, despite these promising findings, research on differential learning using velocity- and acceleration-based methods in youth team sports remains limited, highlighting the need for further studies to facilitate a real understanding of its effects.

Despite the relevance of this method, it is also necessary to consider the specific demands of the sport to optimize the design and implementation of training programs. Although repeated sprint programs are the most representative of linear sprints and game-specific scenarios, sprints are not always performed in this manner due to the multidirectional nature of the sport [21]. Following the principle of specificity to facilitate the transfer of training [22,23], this underscores the need for training methods that prepare for various situations in the game, especially given that each type of training tends to produce trajectory-specific adaptations [24]. In this context, curved sprinting has gained significant interest in recent years. In team sports, this interest has been particularly notable in football, where various observational and experimental studies have been conducted [25,26]. In particular, the neuromuscular differences associated with curved sprints can be quite valuable for preparing athletes for a sport like basketball, which has an open and multidirectional nature [25].

Following the principle of specificity, coaches and strength and conditioning practitioners can develop novel training strategies to equip players for the erratic and intermittent nature of basketball by assessing the combined impact of high motor variability and high-intensity movements with different neuromuscular demands. However, it is important to provide an empirical basis and assess the efficacy of such strategies. Thus, this study aimed to examine the effects of velocity-based curve differential sprinting training on the physical performance of young basketball players. We hypothesized that the differential learning group would show greater improvements in physical performance compared to the control group.

## 2. Materials and Methods

### 2.1. Study Design and Participants

A convenience sample of 24 trained male youth basketball players (mean age: 14.1 ± 1.6 years; average stature: 1.70 ± 0.14 m; typical body mass: 61.4 ± 15.8 kg; basketball experience: 6.0 ± 2.8 years) were recruited for this study. These players were drawn from 3 distinct age groups within the same basketball club academy: U14 (13 players), U16 (7 players), and U18 (4 players) (Table 1). Subjects were divided into the differential training group (n = 13) or the control group (n = 11). The U14 athletes were the only participants with comparable spatial and temporal resources to ensure consistent study conditions throughout the intervention program; therefore, they were included in the differential group. Throughout the experimental phase, spanning from January to March 2024, participants engaged in training sessions three times per week (each lasting 90 min) and competed in one or two matches per week, typically over weekends. To be eligible for the study, players had to be free of injuries and had to have completed all prescribed training sessions in the two weeks leading up to the initial data collection. Participants who missed a testing session or failed to complete at least 90% of the scheduled sprinting training sessions were excluded from the research. Post hoc observed power calculations (G*Power, version 3.1.9.8; University of Düsseldorf; Düsseldorf, Germany) for analysis of covariance (ANCOVA), including two groups and one covariate (α = 0.05, *d* = 0.25), revealed power (β) of 0.21. Written informed consent was obtained from the parents of all participants, with the players providing their assent. The Institutional Research Ethics Committee granted the study approval, following the principles outlined in the Declaration of Helsinki.

A non-blinded experimental controlled trial, featuring two consecutive data collection phases, was utilized to address the research aims. To ensure participants’ familiarity with the physical tests and sprinting exercises, a 20-min familiarization session was conducted a week before the initial data collection. During this session, subjects engaged in unilateral and bilateral CMJs, a 10-m sprint test, and a modified 505 agility test. Following a stochastic approach, the experimental group performed various 20-m curve sprinting exercises. Following the familiarization protocol, all participants underwent testing for CMJ height, 10 m sprint time, and modified 505 change of direction test performance one week later. These tests were chosen based on their established validity and reliability in previous studies involving youth athletes, including basketball players [17,19]. Additionally, their high portability and feasibility in team settings, particularly under time constraints, made them suitable for this study. Additionally, baseline measures including personal (e.g., age, years of basketball experience, team affiliation) and anthropometric (e.g., body mass, height) data were collected at the outset of this testing session. Subsequently, a 12-week intervention phase commenced, during which participants engaged in an additional ~20 min of curved sprinting training program performed twice weekly (on Mondays and Wednesdays), which was embedded as part of their warm-up routine before their basketball-specific practice sessions. Following this, a 2-week retention phase ensued, where no curved sprint training occurred. The control group completed regular basketball practices and matches. Following the 12-week intervention period, participants were instructed to resume their regular training routines without the sprinting program. A week after the final sprinting session (week 13), physical performance measures were reassessed to evaluate changes from baseline to post-intervention. Another week later (week 14), the physical performance tests were then repeated for a final time to assess retention.

### 2.2. Intervention

The experimental group athletes participated in two weekly training sessions during in-court training sessions. All the intervention drills were performed at the beginning of the training session, after the warm-up period, including dynamic stretching and neural activation drills. The differential repeated sprint training comprised curved sprints, similar to those described elsewhere [26]. Participants were required to perform 2 sets of 10 curved sprints on the basketball court, with 30 s of passive recovery between sprints and 3 min of passive recovery between sets. Before each repetition, participants received verbal instruction to perform a different fluctuation of joint velocity (see Table 2) or a combination thereof, selected following the principles of differential learning-based training [12]. No movement variation was repeated within a single training session. In the context of differential learning, athletes with prior experience in diversifying motion geometry parameters were advised to focus on differentiating movement velocity [12]. The training progressed incrementally, introducing one distinct curve sprinting type and starting type (Table 3). Throughout the training program, no adverse events were reported. The control group completed regular basketball practices and matches.

### 2.3. Testing

The testing sessions took place in the familiar setting of an indoor basketball court, the same venue where participants routinely trained during the in-season period. To maintain consistency, participants were instructed to abstain from vigorous physical activity for 24 h before each testing session and fast for at least 2 h beforehand. Both pre- and post-intervention testing sessions began with a standardized warm-up lasting approximately 10 min. This warm-up comprised 3–4 min of running with moderate intensity and dynamic stretching, followed by 6–7 min of bodyweight exercises, including bilateral and unilateral squats, as well as front and side planks. Plyometric exercises, such as unilateral vertical jumps, were also incorporated into the warm-up routine. Following the warm-up, participants were given time (approximately 2–5 min) to hydrate and dry sweat before physical performance measures were taken. To ensure consistency throughout the study period, all tests were conducted in a standardized sequence, adhering to the principles outlined by the National Strength and Conditioning Association for testing order [27]. The testing equipment, measurement protocols, and operators remained constant throughout, with three experienced sport science practitioners overseeing the procedures of both the pre- and post-intervention testing sessions.

*Anthropometrics.* Stature was documented with a commercially portable stadiometer (Tanita BF-522W, Canaxi, Tokyo, Japan), with values rounded to the nearest 0.1 cm. Body mass was approximated utilizing a scale (Tanita BF-522W, Canaxi, Tokyo, Japan), with measurements rounded to the nearest 0.1 kg. All measurements were conducted under the protocols established by the International Society for the Advancement of Kinanthropometry (ISAK) by the same researcher, who possesses an ISAK Level 1 accreditation.

*Jump height.* To assess jump height, participants executed three unilateral (single-leg) and bilateral counter-movement jumps (CMJs) from an upright stance on an infrared contact platform (Optojump, Microgate, Bolzano, Italy). The depth and speed of flexion of the CMJ were self-selected by participants following the Bosco Protocol [28]. The CMJ asymmetry index (CMJ_ASY_) was calculated using the following formula: ASY = 100/Max Value (right and left)* Min Value (right and left)* − 1 + 100 [29]. For subsequent statistical analysis, the highest-performing CMJ was selected from the trials conducted.

*Change of direction performance (Modified 505 agility test).* Participants were instructed to sprint to a mark positioned 5 m from the starting line, execute a 180° change of direction (COD) utilizing either the right or left leg to push off, and return to the starting line, covering a total distance of 10 m [30]. They were required to ensure that their entire foot crossed the line marked on the ground at each turn. The total time for the modified 505 agility test was recorded using photoelectric cells placed at a height of 90 cm and separated by 1.5 m (Witty, Microgate, Bolzano, Italy). Each participant completed two sprints with COD for each side, with a rest period of 2 min between each sprint. Players initiated each trial from a standing staggered position, with their front feet positioned 0.5 m behind the first timing gate.

*Sprint times.* Split times for 10 mand 20 m distances were recorded using single-beam photocell gates positioned 0.9 m above ground level (Witty, Microgate, Bolzano, Italy). Each sprint began from a standing position chosen by the participant, positioned 50 cm behind the first photocell gate, which activated a digital timer upon movement. Participants completed 2 maximal 20 m sprints with 2 min of passive recovery between each sprint. The fastest time achieved for the 10 and 20 m distances was selected for statistical analysis.

### 2.4. Statistical Analysis

Descriptive statistics, such as mean ± standard deviation, were generated for each measure. The reliability of test measures was computed using an average-measures two-way random intraclass correlation coefficient (ICC) with absolute agreement, inclusive of 95% confidence intervals (CIs), and the coefficient of variation (CV). The ICC was interpreted as poor (<0.5), moderate (0.5–0.74), good (0.75–0.9), or excellent (>0.9) [31]. Coefficients of variation were considered acceptable if <10% [32]. In assessing the effects of the 12-week curve sprinting training on physical performance measures for each group, distinct Bayesian Repeated Measures ANOVA (default r scale prior width = 0.5) was used. Considering the baseline value differences between groups, the Bayesian Repeated Measures ANOVA (default r scale prior width = 0.5) using the pre-test value as covariable was used to examine the main effects between training groups (experimental and group) and time (pre-test, post-test, and retention). The Bayesian factor (BF_10_) was then interpreted regarding evidence categories as previously recommended [33]: <$\frac{1}{100}$ = extreme evidence for null hypothesis (H_0_ = no main effects); from $\frac{1}{100}$ to <$\frac{1}{30}$ = very strong evidence for H_0_; from $\frac{1}{30}$ to <$\frac{1}{10}$ = strong evidence for H_0_; from $\frac{1}{10}$ to <$\frac{1}{3}$ = moderate evidence for H_0_; from $\frac{1}{3}$ to <1 anecdotical evidence for H_0_; from 1 to 3 = anecdotical evidence for alternative hypothesis (H_1_); from >3 to 10 = moderate evidence for H_1_; from >10 to 30 = strong evidence for H_1_; from > 30 to 100 = very strong evidence for H_1_; >100 extreme evidence for H_1_. Only those paired comparisons that showed at least strong evidence for supporting H_1_ (BF_10_ > 10) with a percentage error < 10 were considered robust enough to describe significant sprinting training effects. Statistical analyses were performed using JASP software version 0.13.01 (Amsterdam, The Netherlands) and the Statistical Package for Social Sciences (SPSS, v. 28.0 for Mac; SPSS Inc, Chicago, IL, USA).

## 3. Results

All ICCs were excellent (ICC range = 0.91–0.98), and all the CVs were acceptable (CV range = 1.72–6.96%) (Table 4).

**Figure 1:**
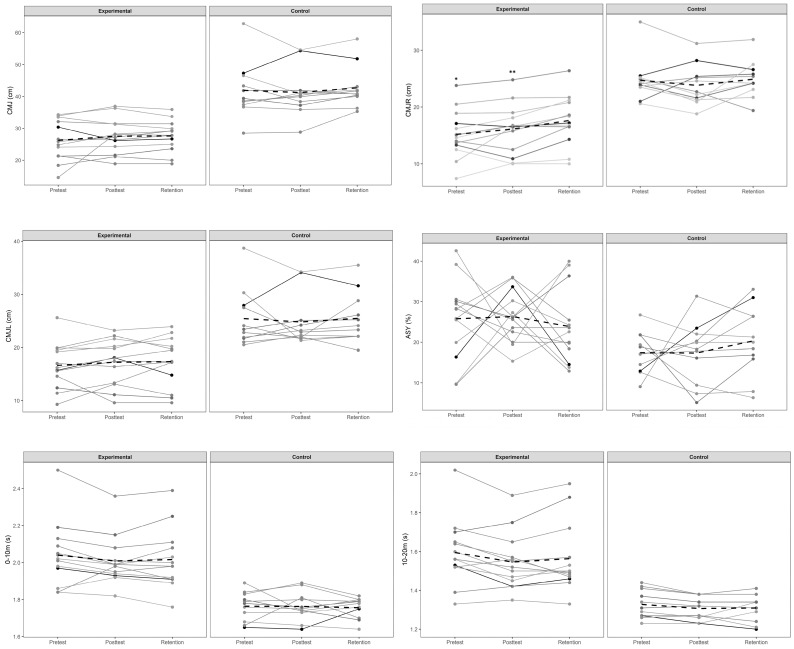

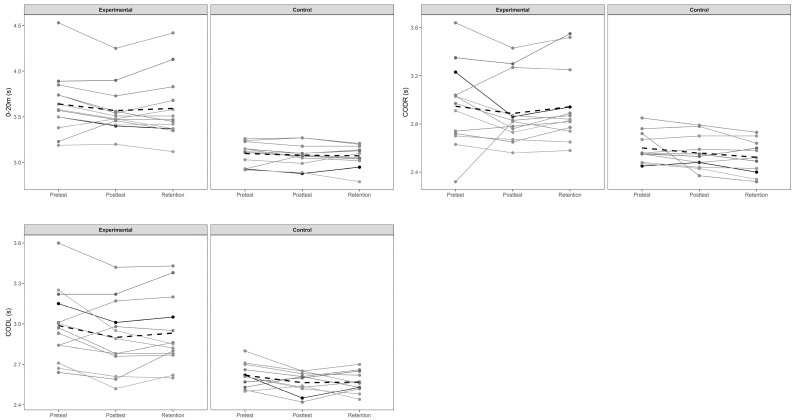
Pre- and post-intervention response comparison for each participant. Note: The dashed line represents the group average. Other lines correspond to the performance of each participant. * corresponds to significant differences between pre-test and retention values; ** corresponds to significant differences between post-test and retention values. Legend: CMJ = counter-movement jump; ASY = bilateral asymmetry; COD = modified 505 agility test.

According to the Bayesian Repeated Measures ANOVA, for the experimental group, time provided the best fit for the data in CMJ height for the right leg (CMJR), 10–20 m sprinting time, and CODL, with a high posterior probability (0.524–0.951) (Figure 1). The highest Bayes factor was found for CMJR (BF_10_ = 19.24), and post hoc comparisons revealed stronger evidence differences between pre-test and retention values (BF_10_ = 10.24), but also between post-test and retention values (BF_10_ = 17.85). In all BF_10_ values observed, a percent error < 10 was shown. The null model proved to be the best fit for the remaining variables for the data. For the control group, the time provided the best fit for the data in CODR and CODL with a high posterior probability (0.524–0.612) (Figure 1). In both tests, post hoc comparisons revealed only anecdotal evidence for H1, with high values observed for differences between pre-test and post-test values (BF_10_ = 2.06–2.27). Also, in this group, the null model proved to be the best fit for the remaining variables for the data. The best Bayesian Repeated Measures ANCOVA model to explain results in CMJL, CMJASY, and 0–10 m sprinting time contained the covariable alone. Moreover, the best model for CMJ and CODR included covariable + group, and the best model for CMJR, 10–20 m and 0–20 m sprinting times, and CODL was time + covariable.

## 4. Discussion

This study aimed to examine the effects of velocity-based differential curve sprinting training on the physical performance of young basketball players. Our results revealed that the 12-week differential curve sprinting training improved physical performance in the experimental group, with the most notable improvement observed in CMJ_R_ height. Differences were observed between pre-test, post-test, and retention values. In contrast, for the control group, the null model appeared to be the best fitting, with only anecdotal evidence of improvement in certain measures, indicating limited training effects from basketball practice alone.

The findings of this study align with highly individualized responses elicited by training stimuli and motor skill practice [11]. Differential learning introduces purposeful variability into motor tasks, enabling individuals to self-organize and develop movement strategies tailored to their unique needs [34]. However, this inherent variability introduces “noise” into the task, potentially requiring extended periods for performers to stabilize their execution strategies. As a result, both positive and negative training outcomes may emerge during such interventions. For instance, in the current study, in participants with right-leg dominance, the intervention might have promoted enhanced force production through the dominant limb, consequently improving CMJ_R_ performance. Conversely, the absence of significant improvements in sprinting performance following the differential curved sprinting intervention may underscore the biomechanical distinctions between linear and curved sprinting. Unlike linear sprints, curved sprints involve distinct biomechanical requirements, such as greater coordination, variable force application, and dynamic balance adjustments. These unique demands may amplify the effects of differential learning, as they require athletes to engage in continuous adaptation and self-organization. While previous studies have identified significant correlations between linear and curved sprint velocities in young soccer players, curved sprinting necessitates asymmetric kinematic adjustments that inherently limit maximal sprinting velocity [26]. Furthermore, the velocities achieved during the differential learning tasks may have been lower than participants’ potential during linear sprints, compounded by the shorter and asymmetric step lengths and ground reaction forces characteristic of curved sprinting [35]. These biomechanical constraints likely explain the lack of positive outcomes in sprint variables, while also aligning with the observed effects on CMJ_R_ performance [36,37].

Within our results, it is interesting to note that improvements were seen for the CMJ_R_ and COD_L_ tasks, which, from the initial outset, highlight discrepancies in the results. Specifically, the CMJ saw improvements in the right leg, but not the left leg. Conversely, there were improvements in COD_L_ time, but not in the right leg, which appears somewhat paradoxical. It could be argued, however, that the penultimate step and thus the forces generated during the right leg in a COD are an inherently important stimulus. Indeed, ref. [38] noted a faster 180° turning performance in conjunction with greater horizontal and vertical ground reaction forces generated during the penultimate step. The resultant forces generated by this penultimate step will be directed posteriorly and superiorly, with muscles in the lower limb acting eccentrically to decelerate the subject’s center of mass. Plausibly, this conforms to the notion of dynamic correspondence, which, within the strength and conditioning domain, represents a criterion-based approach to determining the specificity of a training modality [22,23,39]. Given that the direction in which the forces are acting within the right limb (i.e., along the shank) during the COD task are similar to those observed in the CMJ_R_, it is possible that enhanced capabilities would be observed in both measures.

Again, relating to the specificity of training, the results of our study also found no significant differences in bilateral vertical jump performance. The finding may stem from the discrepancy between the nature of the training task (sprinting) and the assessment method. Although both skills utilize the stretch-shortening cycle, both have obvious differences in biomechanics, including the direction of force production and segment coordination [40]. In contrast with bilateral vertical jumping, sprinting is a unilateral task that requires the coordination of triple extension of the hip, knee, and ankle on one limb, while simultaneously performing triple flexion on the other [41]. Accordingly, the varied training stimulus that characterized the curved running intervention likely encouraged the athletes to make differential use of their limbs, facilitating self-organization based on the specific demands of the task [20]. Therefore, the high variability inherent in the intervention likely contributed to the diverse outcomes observed, which were nonetheless highly specific in nature.

Differential learning, when applied independently, has well-established effects, particularly in the areas of neuromuscular adaptation and brain activity. For instance, studies have shown that performing tasks such as jumping rope or serving in badminton with varying techniques leads to increased brain activity in specific regions and frequencies, indicative of enhanced motor learning [42,43]. Similarly, strength training exercises based on differential learning principles have been associated with greater activation of major muscle groups compared to traditional methods, underscoring the unique benefits of variability in training [44]. Additionally, incorporating differential learning into repeated sprint training has demonstrated notable improvements in locomotor performance, including sprinting, jumping, and agility [17,19]. Therefore, the outcomes of the program in the current study are likely the result of a synergistic interaction between curved sprinting and the differential learning approach. These findings highlight the capacity of differential learning to drive positive adaptations and suggest that the combined methodology appears to offer unique benefits. However, introducing novel motor skills through a combination of curved sprinting and differential learning may also initially disrupt motor patterns, leading to temporary asymmetries. These changes can be attributed to cortical reorganization, as the brain adjusts to new training demand asymmetries [45]. Interestingly, these asymmetries tend to decrease during retention periods, suggesting that the nervous system stabilizes motor patterns over time. This stabilization process may involve minimizing unnecessary asymmetries while optimizing overall movement efficiency, highlighting the long-term benefits of combining differential learning and curved sprinting.

Despite the interesting findings of our study, an obvious limitation is the lack of a true control group that allows for isolating the effects of the approach used in this study. The control group in this study recurrently participated in basketball training and games, which may have influenced the observed differences between groups. In addition, a curved sprint group versus a curved sprint combined with a differential learning group may have provided richer insights. In all instances, it is necessary to take into account that not only the duration of the training program but also the retention time can have a decisive influence on the results found since the adaptations in these two phases are often time-dependent. Another limitation includes the use of a convenience sample, which may limit the representativeness of the participants. The reliance on a readily accessible group, rather than a randomly selected sample, increases the risk of sampling bias and may lead to group-specific characteristics influencing the observed outcomes. This highlights the need for caution when interpreting the results and reinforces the importance of future studies employing more robust sampling methods to strengthen the validity of the findings.

## 5. Conclusions

This study demonstrated that velocity-based differential curve sprinting training effectively enhances physical performance in young basketball players, particularly in improving CMJ_R_ and COD_L_. The differential learning approach, emphasizing variability and adaptability, proved to be a valuable training method, aligning with the dynamic and multidirectional demands of basketball. The improvements observed in the experimental group highlight the potential of introducing variability through curved sprinting, which prepares athletes for sport-specific scenarios requiring adaptability and neuromuscular coordination. The unique nature of curved sprints, combined with differential learning principles, may foster individualized adaptations, benefiting physical development even in resource-limited settings. Nonetheless, some athletes did not respond to the intervention. Although improvements in sprinting performance were not the primary focus of this study, the findings indicate the influence of task-similar training strategies to maximize overall athletic potential. Future research can expand on these findings by exploring complementary training interventions to further optimize outcomes. Overall, this study emphasizes the value of velocity-based curve differential sprinting as a practical and effective tool for developing youth basketball players’ physical capabilities and game-specific performance.

## Figures and Tables

**Table 1:** Anthropometric characteristics and training experience of experimental and control groups. Values presented as mean ± standard deviation.

Group	Age (years)	Height (cm)	Body Mass (kg)	Training Experience (years)
**Control**	15.6 ± 1.1	178.4 ± 11.9	71.3 ± 11.3	7.0 ± 2.9
**Experimental**	12.9 ± 0.7	162.9 ± 14.0	53.0 ± 14.4	5.1 ± 2.4

**Table 2:** Training program variations.

Component	Fluctuations	Component	Fluctuations
**Body part**	Upper body slightly faster than lower body	**Body joint**	Right knee slightly faster than left
	Upper body largely faster than lower body		Left knee slightly faster than right
	Right side slightly faster than left side		Right knee largely faster than left
	Left side slightly faster than right side		Left knee largely faster than right
	Right side largely faster than left side		Ankles slightly faster than hips
	Left side largely faster than right side		Ankles largely faster than hips
	Upper body faster than lower body		Ankles slightly faster than knees
	Right lower body slightly faster than left lower body		Ankles largely faster than knees
	Left lower body slightly faster than right lower body		Knees slightly faster than hips
	Right lower body largely faster than left lower body		Knees largely faster than hips
	Left lower body largely faster than right lower body		Knees slightly faster than ankles
**Body joint**	Right arm slightly faster than left arm		Knees largely faster than ankles
	Left arm slightly faster than right arm		Hips slightly faster than knees
	Right arm body largely faster than left arm		Hips largely faster than knees
	Left arm largely faster than right arm		Hips slightly faster than ankles
	Right ankle slightly faster than left		Hips largely faster than ankles
	Left ankle slightly faster than right	**Phase**	Eccentric phase slightly faster than concentric phase
	Right ankle largely faster than left		Eccentric phase largely faster than concentric phase
	Left ankle largely faster than right		Concentric phase slightly faster than eccentric phase
	Right hip slightly faster than left		Concentric phase largely faster than eccentric phase
	Left hip slightly faster than right	**Full movement**	Increasing velocity
	Right hip largely faster than left		Decreasing velocity
	Left hip largely faster than right		Interchangeable velocity

**Table 3:** Training program schedule.

Weeks	1–3	4–6	7–9	10–12
**Starting types**	2-point	3-point	4-point	Flying
**Sprinting type**	Curve sprinting	Double curve sprinting	180° curve sprinting	Curve sprinting

**Table 4:** Reliability data for test variables. Data are presented as values with lower and upper confidence limits.

Test Variables	ICC (95% CL)	CV (%) (95% CL)
CMJ (cm)	0.98 (0.96; 0.99)	4.12 (2.82; 5.41)
CMJ_R_ (cm)	0.98 (0.96; 0.99)	6.85 (4.88; 8.82)
CMJ_L_ (cm)	0.96 (0.92; 0.98)	6.96 (5.04; 8.88)
0–10 m (s)	0.98 (0.95; 0.99)	1.83 (1.33; 2.32)
10–20 m (s)	0.98 (0.95; 0.99)	2.10 (1.47; 2.73)
0–20 m (s)	0.98 (0.96; 0.99)	1.72 (1.21; 2.23)
COD_R_ (s)	0.91 (0.79; 0.96)	3.09 (1.66; 4.52)
COD_L_ (s)	0.97 (0.92; 0.99)	2.09 (1.51; 2.68)

## Data Availability

The data that support the findings of this study are available from the corresponding author, J.A., upon reasonable request.

## Abbreviations

The following abbreviations are used in this manuscript:

BF_10_	Bayes factor
CI	Confidence interval
CMJ	Counter-movement jump
CMJASY	CMJ asymmetry index
CMJL	Counter-movement jump (left leg)
CMJR	Counter-movement jump (right leg)
COD	Change of direction
CODL	Change of direction (left leg)
CODR	Change of direction (right leg)
CV	Coefficient of variation
H0	Null hypothesis
H1	Alternative hypothesis
ICC	Intraclass correlation coefficient
